# Aging and urinary control: Alterations in the brain–bladder axis

**DOI:** 10.1111/acel.13990

**Published:** 2023-09-22

**Authors:** Cara C. Hardy, Ron Korstanje

**Affiliations:** ^1^ The Jackson Laboratory Bar Harbor Maine USA

**Keywords:** aging, molecular biology of aging, neuroscience

## Abstract

Age‐associated alterations in bladder control affect millions of older adults, with a heavy burden added to families both economically and in quality of life. Therapeutic options are limited with poor efficacy in older adults, lending to a growing need to address the gaps in our current understanding of urinary tract aging. This review summarizes the current knowledge of age‐associated alterations in the structure and function of the brain–bladder axis and identifies important gaps in the field that have yet to be addressed. Urinary aging is associated with decreased tissue responsiveness, decreased control over the voiding reflex, signaling dysfunction along the brain–bladder axis, and structural changes within the bladder wall. Studies are needed to improve our understanding of how age affects the brain–bladder axis and identify genetic targets that correlate with functional outcomes.

## Graphical abstract

The aged brain–bladder axis experiences alterations in both the CNS‐sphere of influence and the bladder sphere of influence. Brain changes include an increased reliance on an intact CNS, decreased precision of CNS control over the bladder reflex, and altered afferent signaling. Bladder‐level alterations include decreased drug responses, increased collagen deposition, and decreased volume sensitivity, though muscle strength is preserved.

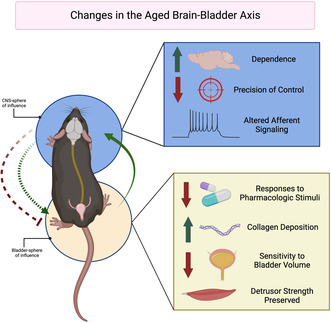

## 1 INTRODUCTION

Advancements in modern medicine have significantly increased lifespan; however, improvements in healthspan are just beginning to be addressed. As we age, the functionality of many of our organ systems begins to decline (Liberale et al., 2022; Noordmans et al., 2015; Poulose & Raju, 2014). The lower urinary tract (LUT)—which consists of the urinary bladder and the urethra—experiences age‐related functional declines in both humans and animal models (Siroky, 2004).

### 1.1 Neural control of micturition

**FIGURE 1:**
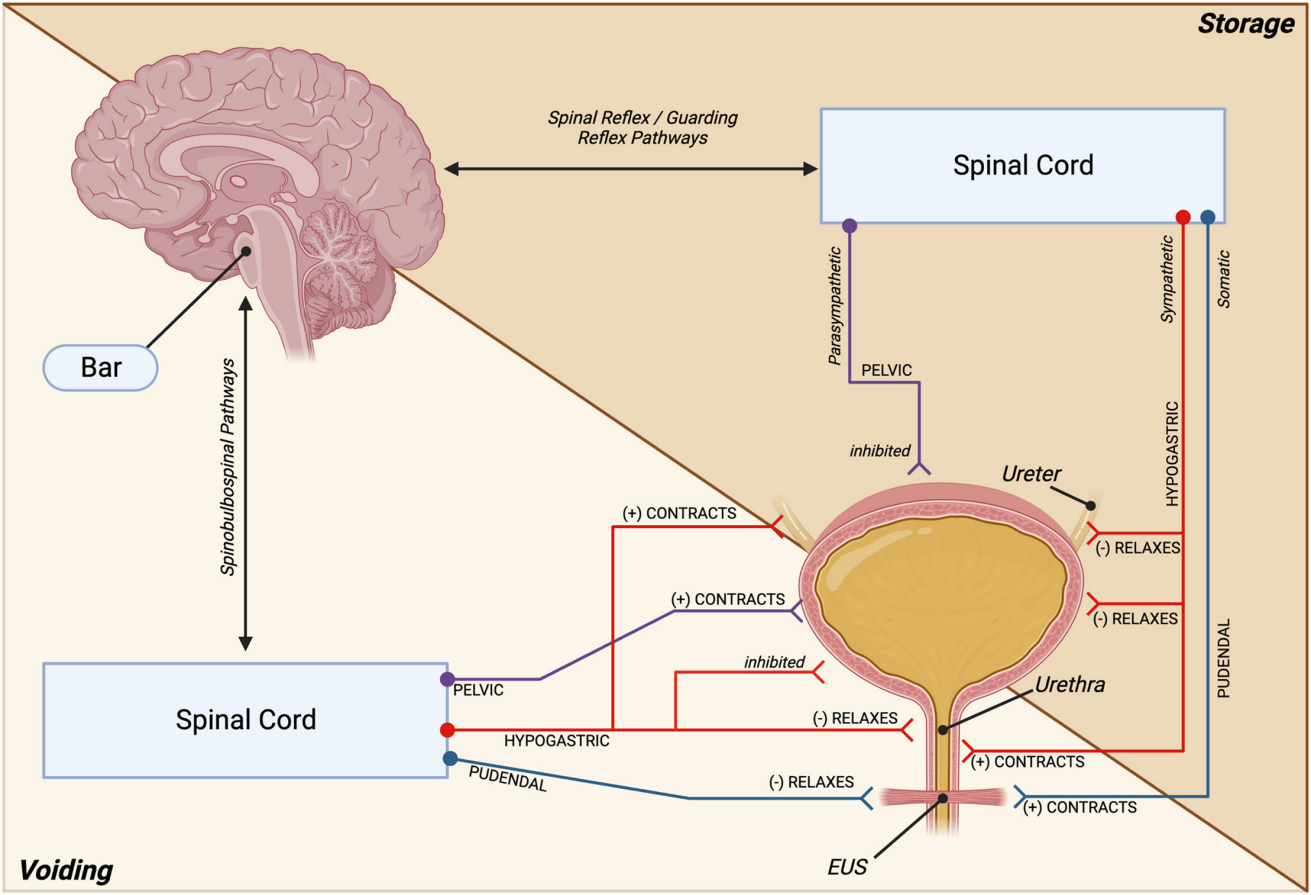
Neural mechanisms of storage and voiding. *Voiding*: Emptying of the bladder is initiated by coordinated contraction of the detrusor via the pelvic nerve and cholinergic signaling (purple) and simultaneous relaxation of the urethra via the hypogastric nerve and adrenergic signaling (red) and external urethral sphincter (EUS) via the pudendal nerve and cholinergic signaling (blue). Adrenergic relaxation mechanisms are inhibited in the detrusor. The ureteral sphincter is also closed, preventing backflow of urine into the kidneys during bladder emptying. Activation of these peripheral nerves is through the spinal‐bulbospinal pathways relaying signals from Bar. *Storage*: Urine storage occurs when sympathetic adrenergic signaling from the hypogastric nerve relaxes the bladder wall and ureteral sphincter while contracting the bladder neck. Somatic signaling from the pudendal nerve maintains EUS tonus, ensuring the sphincter stays closed during filling. Parasympathetic signaling of the pelvic nerve is inhibited during urine storage. Spinal reflex and guarding pathways govern the control over urine storage.

Micturition, urination, or voiding—terms used interchangeably to describe the process of expelling urine from the bladder—should ideally be able to be performed involuntarily and voluntarily. While our understanding of the aged LUT is still incomplete, many of the underlying mechanisms permitting urinary storage and voiding have been documented over the last several decades. The hallmark of successful LUT function is to store urine at low pressure, which prevents renal reflux through the ureters and subsequent kidney damage. Neural control over the urinary tract is governed both consciously and unconsciously (de Groat, 2006; Fowler et al., 2008). The micturition reflex is an *involuntary* process by which bladder volume is relayed to the central nervous system (CNS), specifically the periaqueductal gray (PAG) where it is processed and relayed to Barrington's nucleus (Bar, also called the Pontine Micturition Center, PMC) in the pons of the brainstem (Figure 1). This pressure information will eventually reach the Threshold Pressure, which triggers micturition. Conscious control by the prefrontal cortex allows for *voluntary* voiding behavior, as relaxatory adrenergic signaling can be initiated to prolong urine storage in instances of inconvenience (such as waiting for the next rest‐stop on a long drive) and as contractile muscarinic signaling to initiate urination in instances of convenience (such as using the restroom before getting on a flight, despite not feeling the “need to go”). The descending brain‐to‐bladder signaling relay is largely controlled by Bar. Fowler et al. and de Groat provide excellent resources for understanding the basis of neural control over micturition in greater depth (de Groat, 2006; Fowler et al., 2008).

### 1.2 Tissue‐level control of voiding and storage mechanisms

**FIGURE 2:**
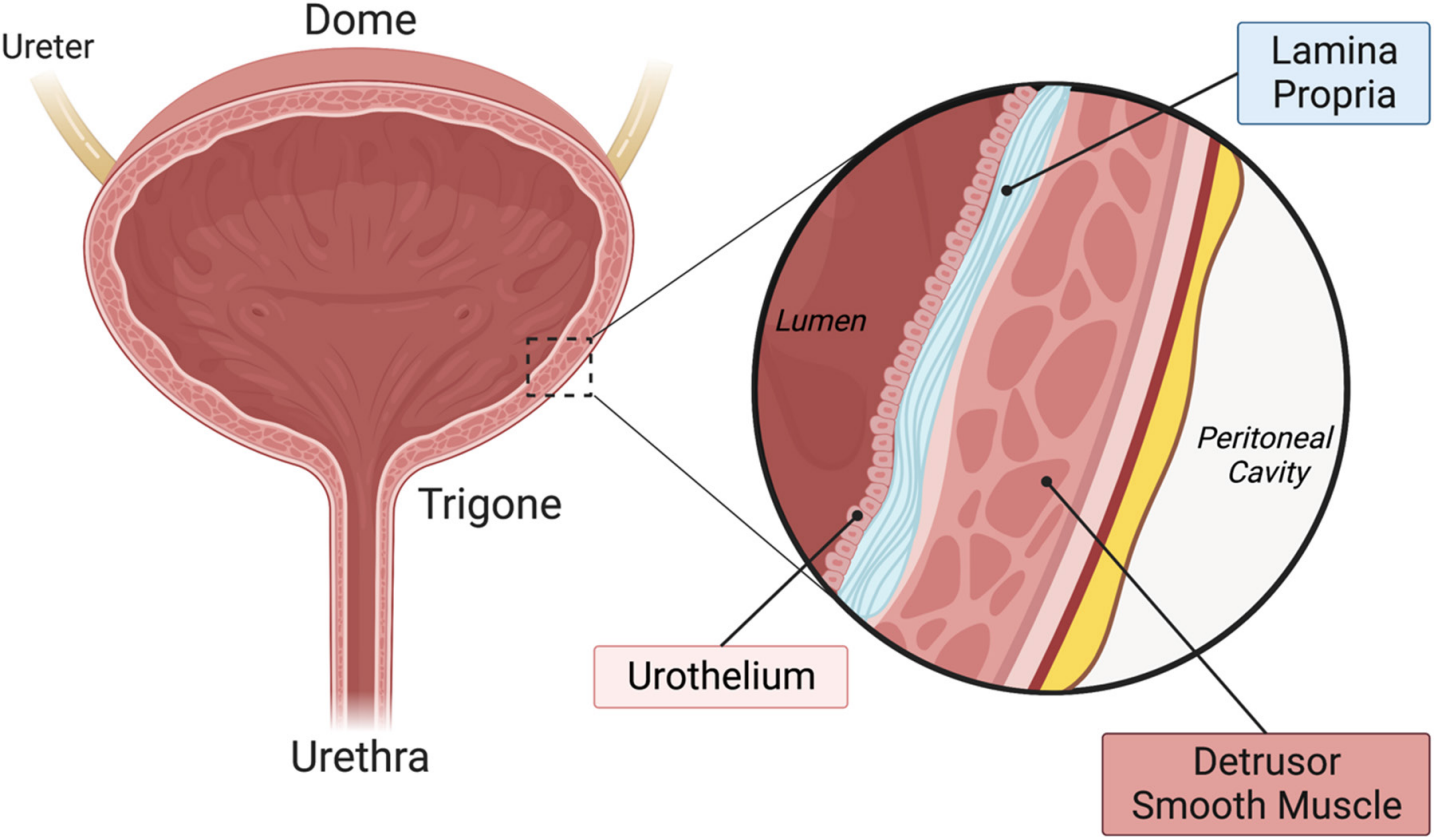
Bladder wall architecture. (Left) Regions of the bladder. The bladder reservoir is depicted here as distended (full), with the urine not pictured. Broad regional classifications of tissue location (particularly in the instance of biopsies) as coming from the dome or the trigone, which each have structural and molecular differences. The dome or “body” of the bladder is rich with vascularization and demonstrates increased expression of receptor transcripts, including muscarinic, P2X, ASIC, and NK receptors compared to that of the trigone. The trigone is a triangular region spanning from the urethra to the ureter inputs into the bladder lumen. Unlike the dome, the trigone stretches very little, as it has a higher composition of connective tissue and increased expression of cell‐adhesion and tight junction. (Right) Cross‐sectional view of the bladder wall. The lumen—where urine is stored—is lined with uro‐epithelial cells, called the urothelium, acting as a distensible, water‐tight signaling mechanism. The lamina propria is the layer of connective tissue between the urothelium and smooth muscle layers. The detrusor smooth muscle comprises most of the bladder wall, contracting to expel urine and relaxing to store urine.

**FIGURE 3:**
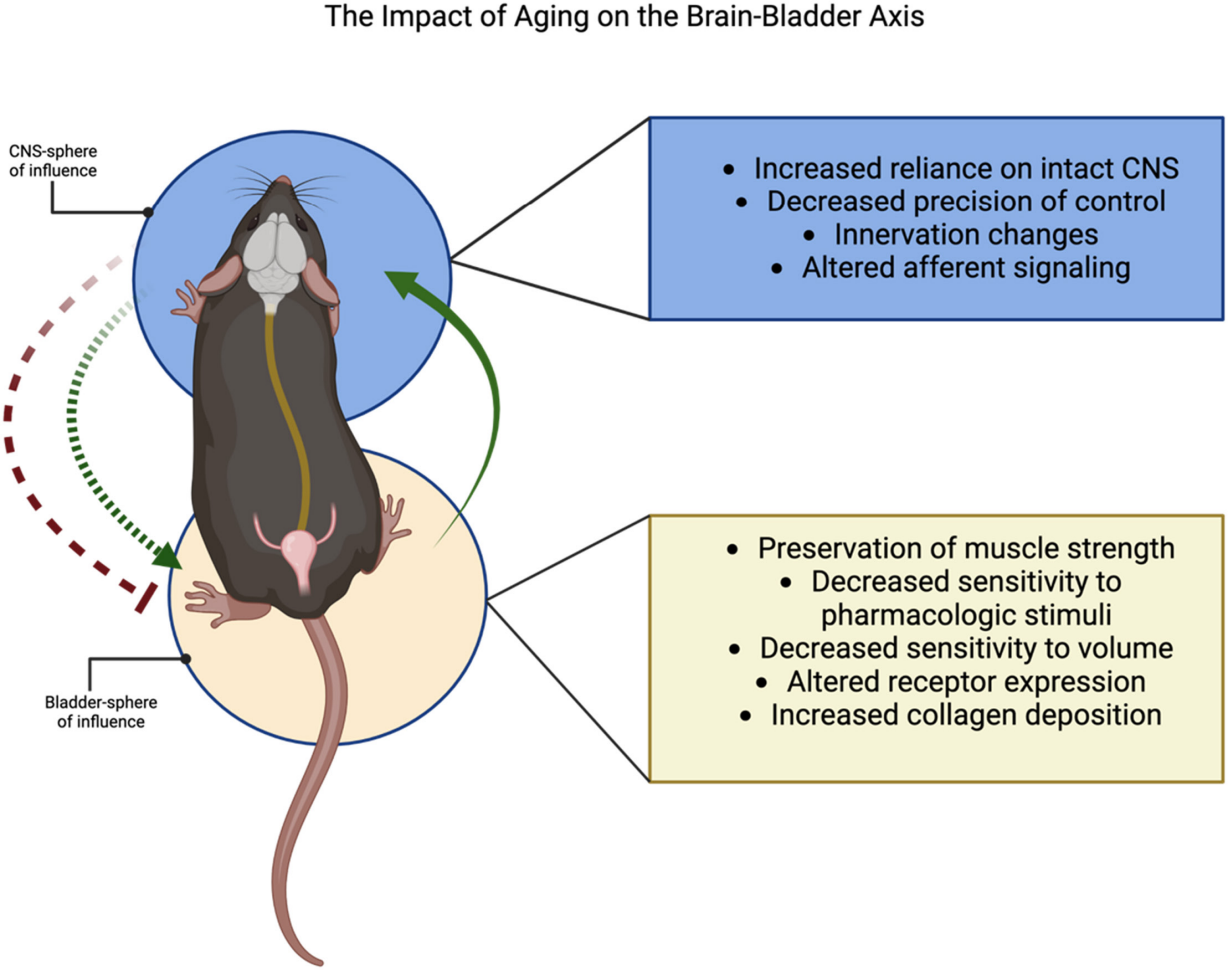
Age‐associated changes along the brain–bladder axis. (Left) A depiction of the brain–bladder axis in mouse. Solid green arrow depicts the continuous afferent data stream ascending to the CNS. The dashed lines are indicative of efferent signaling pathways: Parasympathetic activation of the muscle (green) results in bladder contraction, while sympathetic signaling (red) delivers instructions to relax the bladder muscle and accommodate increasing bladder volumes. (Right) Summarized age‐associated changes along the brain–bladder axis impacting the CNS control over micturition (blue) and bladder tissue‐specific changes (yellow).

In addition to neural control mechanisms, the bladder tissue is also subject to local influences of the muscle and epithelial layers. The bladder wall itself is comprised predominantly of smooth muscle, connective tissue, and an epithelial barrier (Figure 2). The most prominent layer of the bladder wall is the detrusor smooth muscle, which relaxes due to adrenergic signaling and contracts with muscarinic signaling initiated by Bar. The urothelium is the mechanosensitive epithelial barrier which secretes a variety of inhibitory and excitatory factors, such as NO and ATP. As the bladder lumen (where urine is stored) expands, the urothelium distends, initiating this signaling cascade (Birder & Andersson, 2013). Between the detrusor and urothelial layers lies a network of connective tissue: the lamina propria. Factors released from the urothelium act on dorsal root ganglion (DRG) neurons—sensory fibers projecting from the spinal cord that relay bladder volume back to the CNS—providing influence over afferent signaling and thus the micturition reflex (Andersson & McCloskey, 2014). The balance of excitatory and inhibitory factors acts to set detrusor tonus—how stretchy or stiff the bladder wall is—to achieve urine storage at low pressure. The interplay of local signaling between the layers of the bladder wall and neuronal signaling work collectively to set the detrusor tension, adapting as needed to accommodate increasing volumes or empty the bladder reservoir. Loss of control over urinary function can be attributed to maladaptive changes along the brain–bladder axis: alterations in the mechanisms of control via the CNS sphere of influence, the bladder tissue‐level sphere of influence, or a combination of both (Figure 3).

## 2 CHANGES WITH AGING ALONG THE BRAIN–BLADDER AXIS

The brain–bladder axis acts as an information superhighway in which sensory information from the bladder is relayed, interpreted, and acted upon (Figure 1). This highly adaptive system works in concert to communicate bladder volume changes and pathologic/noxious stimuli to the CNS while simultaneously adjusting the compliance of the bladder and the tonus of the external urethral sphincter (EUS) accordingly. Under homeostatic norms, specific challenges along the brain–bladder axis can be accommodated. For example, a urinary tract infection (UTI) often results in pain symptoms, as well as feelings of frequency and urgency (Foxman, 2010). In most cases, though, one can expect to maintain continence during a UTI—assuming the integrity of the brain–bladder axis is intact. The aged milieu disrupts homeostasis systemically down to the cellular level (Zhang et al., 2019). As increased demands are placed upon the brain–bladder axis, the adaptive range in which stressors can be accommodated while still maintaining function begins to decrease. While a UTI in young individuals may result solely in discomfort, older individuals will have a much higher likelihood of experiencing incontinence as well (Moore et al., 2008).

The brain–bladder axis can be experimentally interrogated at multiple levels, depending on the question being asked. The micturition reflex is frequently studied using cystometry, an experimental technique analogous to human urodynamics. Under urethane anesthesia, cortico‐thalamic connections are interrupted (Huh & Cho, 2013), and brainstem‐mediated intravesicular bladder pressure can be measured through the surgical insertion of a catheter through the dome of the bladder, enabling the assessment of the micturition reflex without cortical interference (Smith & Kuchel, 2010) (Box 1). Voiding behavior, a measure of cognitive control over the micturition reflex, can be accessed via voiding spot assays (Hill et al., 2018) (Box 1), as well as awake, free‐moving cystometry (Fraser et al., 2020). Alterations in urinary physiology at the tissue level are commonly investigated through pharmacomyography—also called strip studies—in which ex vivo preparations of bladder tissue are exposed to pharmacologic and electrical stimuli (Kullmann et al., 2014) (Box 1). Strip studies provide an opportunity to look at tissue level responses without the influence of the CNS.

BOX 1 Experimentally interrogating the brain–bladder axis. The bladder is highly reliant upon the CNS, and there are several different methodologies that can be employed to study different aspects of the brain–bladder axis. Void spot assays (VSAs) are useful to determine voiding behavior in awake, freely moving animals and can give insight into the cognitive component of urinary physiology (top panel, blue). Mice are place in a cage lined with filter paper, and the urine spots are UV imaged. Spot size, the number of spots, volume/area of the voids, and corner vs central preference all give insight into bladder function. Young animals, for instance, tend to have more large voids than small voids, and in general tend to urinate in the corners/periphery of the enclosure due to thigmotaxis—a phenomenon in which rodents tend to stay along the edges for safety as opposed to venturing into the open middle (Simon et al., 1994). Old animals, on the contrary, tend to have larger numbers of small spots (believed to be evidence of urine leakage), and they tend to void more in the center as well. Cystometry encompasses both the CNS and bladder spheres of influence, providing detailed information on the voiding reflex coordination of CNS signaling and that of the bladder. Bladder pressure is measured through a catheter inserted into the dome of the bladder, and flow rate/volume voided measured through a force transducer underneath the urethra. Generally, this is performed under urethane anesthesia, which enables the assessment of the reflex without cognitive input (middle panel, green). Pharmacomyography, also called bladder strip studies, is used to understand mechanistically what contributes to detrusor smooth muscle contraction or relaxation. Drugs or electrical stimulation are used to interrogate specific pathways and can give insight into the mechanisms behind the functional observations seen in cystometry and VSAs (bottom panel, yellow). 
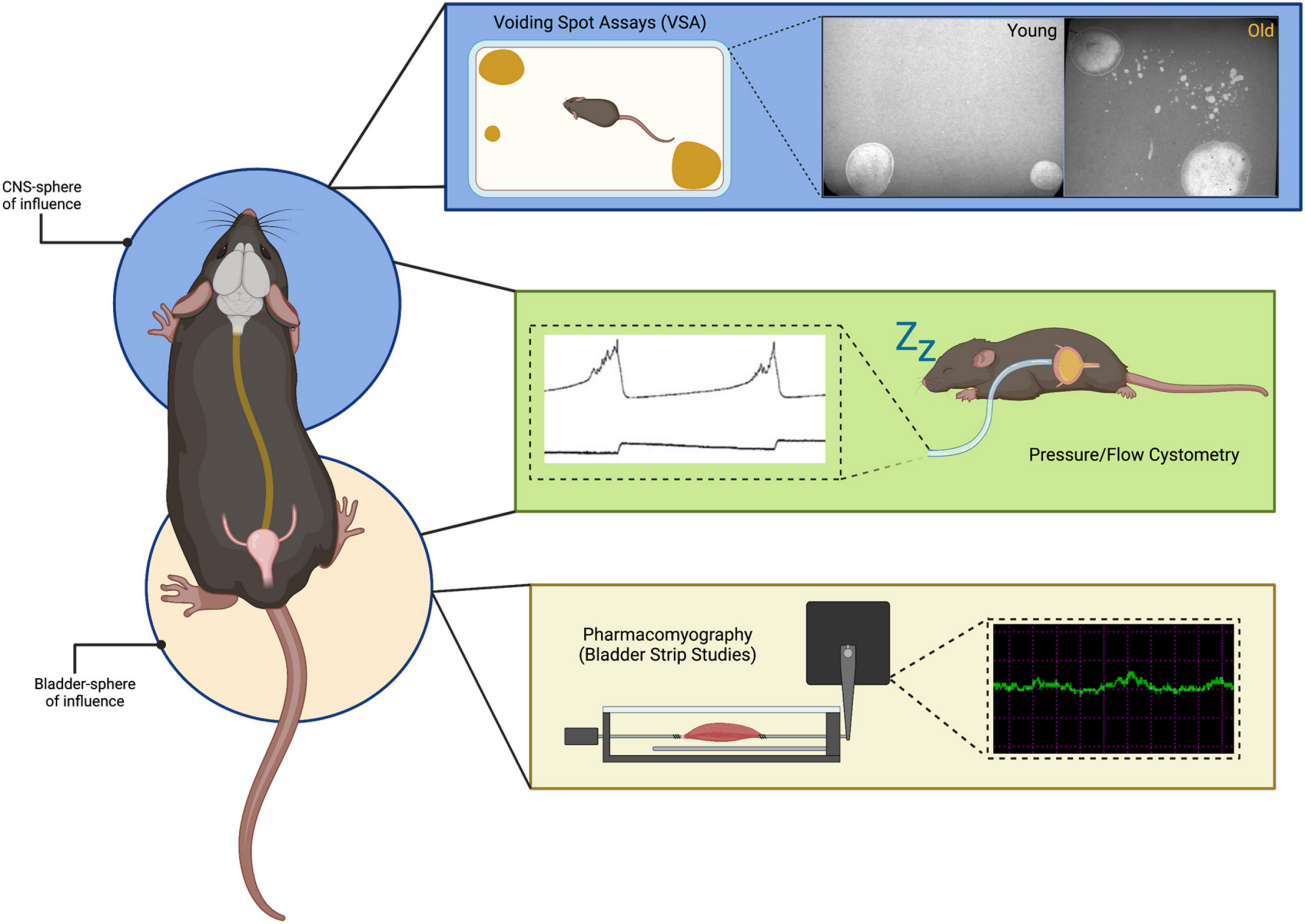

### 2.1 CNS integrity is essential for urinary function and is compromised with aging

Though the biophysical properties and limitations of the bladder are undoubtedly important for urinary control, in the context of aging there is an increased reliance upon CNS integrity for maintaining normal function (Smith, DeAngelis, & Simon, 2015). Altering CNS function through isoflurane anesthesia results in cystometric curves indistinguishable from that of deceased animals, demonstrating the reliance of compliance on CNS integrity during bladder filling (Smith et al., 2012a). In the aged mouse, there is a loss of sensitivity to bladder volume—often regarded as a model of detrusor underactivity—that represents a lack of systemic awareness of how much urine is present in the bladder reservoir. It is unknown if this phenomenon is due to decreased afferent firing in response to volume changes and a subsequent loss of information from afferent signaling, or if this is the result of improper neural processing. If the latter is true, afferent firing rates would be unaffected, but threshold pressure—the point at which the system switches from storage to voiding—would be temporally shifted. As micturition interval—the time between voiding events—is known to increase with aging (Hardy et al., 2019; Kullmann et al., 2014), afferent signaling changes with aging warrants further study.

Conversely, investigators have reported increased volume sensitivity in non‐anesthetized mice that is paired with increased afferent firing rates. One study found that aged mice exhibited increased voiding frequency and afferent activity compared to young animals. These results suggest that the hyper‐excitable phenotype observed models the detrusor overactivity and increased urinary frequency observed in some older adults, which may be the result of increased sensitivity to volume through enhanced purinergic signaling (Daly et al., 2014). There have been conflicting reports as to whether bladder innervation is increased (Schueth et al., 2016), decreased (Nakayama et al., 1998), or maintained with aging (Mohammed & Santer, 2002), further complicating the interpretation of these findings. The dichotomy of these observations supports the hypothesis that aging in the mouse brain–bladder axis is a heterogenous process, as is observed in humans: Some bladders become more sensitive, some become less sensitive, but in either case, the precision of control over this system diminishes and the likelihood of aberrant responses increases.

### 2.2 Barrington's nucleus: Glutamatergic signaling is negatively impacted by aging

To date, there are no published studies specifically addressing the impact of aging on Bar. Despite this gap in our knowledge, some anecdotal findings may serve as useful resources for hypothesis generation. Bar neurons are glutamatergic, and it has been well documented that glutamatergic neurons are susceptible to aging: Glutamatergic transmission is decreased, and structural changes to the glutamatergic neurons are observed, including decreased size, shortened axons, and fewer dendritic branches (Drachman, 2006; Gasiorowska et al., 2021). As Bar has subsets of CRH+ neurons responsible for contraction (Pavcovich & Valentino, 1995), age‐associated changes in CRH signaling could impact urinary function. CRH expression, as well as CRH‐BP (CRH‐binding protein) expression, has been reported to decreased in the amygadala of aged (24 months) Fischer 344 rats compared to 4‐ and 12‐month old animals (Xiao et al., 2006). It is unknown how aging impacts CRH expression in Bar, but alterations in this expression would likely lead to decreased contractility mechanisms.

### 2.3 The aging brain: atrophy, vascular deficiencies, and dysfunction at the cellular level

Brain aging has been well documented in older adults: physical alterations occur, such as the shrinkage of cortical areas involved in memory and complex decision making tasks, as well as changes in signaling and plasticity, as demyelination and decreased efficiency of cell‐to‐cell communication between neurons and glia have been observed in both human and animal models (Blinkouskaya et al., 2021; Radulescu et al., 2021). As successful urinary function is highly contingent upon the state of the CNS and communication between the brain and bladder, it is safe to assume the age‐associated changes in the brain will directly impact urinary control, though the precise mechanisms by which this occurs have not been extensively studied.

One of the major challenges in understanding the role of brain aging in urinary dysfunction is due to the uniqueness of the individual: It is exceedingly difficult, even when assessed longitudinally, to identify subtle changes in the brain that directly correlate with dysfunction (Scahill et al., 2003). Despite this challenge, hallmark changes in the aging brain have been identified. Tissue atrophy, vascular deficits, inflammation, and cellular changes—such as decreased metabolism, impaired signaling, and diminished autophagy—have all been reported (Peters, 2006). Function brain imaging (fMRI) studies have corroborated that decreased brain volumes, vascular dysfunction, and metabolic decline contribute to urinary phenotypes such as detrusor overactivity, detrusor underactivity, and incontinence (Andersson et al., 2017; Smith, Kuchel, & Griffiths, 2015). Though the aforementioned changes are by no means a complete list of all age‐related changes in the CNS, these parameters in particular may have important implications for the functionality of the urinary system.

### 2.4 Loss of brain volume contributes to urinary dyfsunction

Volume loss of both grey and white matter are some of the most prevalent features of the aging brain (Blinkouskaya et al., 2021; Peters, 2006). Cortical thinning is also a hallmark feature of the aging brain, with simultaneous expansion of the ventricles. Neuron loss and subsequent atrophy have been shown to correlate with cognitive decline (Fjell & Walhovd, 2010), and likely impact the ability of older adults to consciously govern the voiding reflex. Brain lesions, including those associated with neurodegenerative disorders, of the forebrain often result in incontinence, while lesions of the brainstem and spinal cord are generally associated with urinary retention (Tish & Geerling, 2020). Ventricular enlargement is not just due to loss of neurons but can in fact be causal. Decreased cerebrospinal fluid (CSF) circulation in older adults compresses the surrounding brain tissue and vasculature, leading to neuronal loss and cognitive deficits (Todd et al., 2017). Loss of neuronal tissue undoubted disrupts connections between brain centers implicated in voluntary and reflexive urinary control, contributing to voiding dysfunction in older adults.

### 2.5 Vascular deficits in the CNS impact downstream function

Vascular deficits, including damage from stroke, are also associated with brain aging, particularly in cases of dementia. In patients with vascular cognitive impairment, the second most common cause of dementia, urinary dysfunction is often experienced as well (Zhao et al., 2022). Additionally, modeling of cerebral hypoperfusion (ischemic injury, such as stroke) in rats results in bladder dysfunction (Liang et al., 2015). Vascular deficits in the brain are often reflective of systemic vascular issues (Cortes‐Canteli & Iadecola, 2020) and can likely contribute to bladder ischemia as well.

### 2.6 Dysfunctional cellular processes in the aging bladder

Damage from decreased perfusion and lesions (such as amyloid plaques and white matter hyperintensities) directly impacts cellular function, leading to dysfunction in fundamental cellular processes such as autophagy, mitochondrial function, and inter‐/intra‐cellular communication. The brains of aged animals exhibit deficiencies in mitochondria, namely increased production of reactive oxygen species (ROS), decreased mitochondrial membrane potential, and impaired electron transfer (Navarro & Boveris, 2010). Production of cyto‐toxic ROS can induce further damage, contributing to increased cellular senescence in the brain and an impaired extracellular environment, disrupting cell‐to‐cell communications essential for neuronal transmission and bladder function downstream. Impaired autophagy also aids in this phenotype: The inability to effectively clear cellular byproducts can lead to neuronal death, providing one of many cellular mechanisms responsible for the structural changes and atrophy observed in the aging brain (Plaza‐Zabala et al., 2017). The increase in microgliosis and inflammation of the aged CNS cannot be ruled out as potential contributors to urinary dysfunction, as an inflamed extracellular milieu will impair neuronal transmission and contribute to neurodegeneration (Allen et al., 2023; Muzio et al., 2021).

### 2.7 Spinal circuitries are negatively impacted by aging

Spinal reflex pathways have been shown to be negatively impacted by aging. Signaling efficiency along the spinal reflex pathways is decreased, and the system becomes less adaptive (Geertsen et al., 2017). One group reported improvements in urinary retention following the targeted removal of senescent cells in a spinal cord injury mouse model (Paramos‐de‐Carvalho et al., 2021). Although this is an injury model and not a model of aging, improvements after the clearance of senescent cells from aged animals have been reported in other systems (Wang et al., 2021). Similar pathologies to those of the aging brain have been described in the spinal cord: Impaired autophagy, neurodegeneration, vascular changes, and low‐grade inflammation are all reported in aged mice (Piekarz et al., 2020; Ward et al., 2022). It is likely that these changes contribute to dysfunction in communication along the brain–bladder axis.

It is currently unknown whether spinobulbospinal or guarding reflex pathways are impacted equally, or if one is more susceptible to aging associated changes over the other. Based on known aging changes (such as decreased receptor density/sensitivity), it is highly probable that both pathways are impacted to some degree, though further studies are necessary to fully understand the breadth of these changes, as well as how subjective these changes are to each individual. For example, detrusor underactivity could indicate spinobulbospinal pathway pathophysiology, implicating that contractile signaling mechanisms are adversely altered. Conversely, detrusor overactivity may not have spinal‐bulbospinal pathway dysfunction, but rather impairments in the guarding reflex. While tempting to consider spinobulbospinal pathway dysfunction in instances of increased detrusor activity, the inability to appropriately signal storage (and thus, the inability to accommodate increasing volumes of urine while maintaining relatively low intravesicular pressures) could lead to premature voiding signaling from the CNS due to high intravesicular pressures despite relatively low bladder volume. Animal models are a necessary next step to elucidate the effect of aging on these pathways. Pharmacologic stimulation of nerve preparations of hypogastric, pelvic, and/or pudendal nerves from aged and young/mature animals will undoubtedly answer many of these questions, providing insight into which specific pathways are most severely impaired in aging, and which ones—if any—are preferentially spared. Neurotransmitter synthesis is known to be generally impaired with aging, as is the case with dopamine and serotonin (Peters, 2006). However, desensitization/decreased expression of receptors may be driving increased production of certain neurotransmitters. In humans, aging has been associated with increased presence of acetylcholine and decreased ATP (Yoshida et al., 2001). Proteomics and metabolomics studies may be a necessary next step to uncovering which neurotransmitters are most susceptible to aging along the brain–bladder axis.

### 2.8 Bladder aging: changes in peripheral structure and function

Treatment of urinary dysfunction in older adults has largely been bladder‐centric: therapeutics predominantly target the detrusor muscle. There is a lack of therapeutic success seen in drugs targeting adrenergic and cholinergic mechanisms in patient populations. Anticholinergic drugs, for example, are largely ineffective and are not generally recommended in older patients due to the cognitive side effects (Risacher et al., 2016). The reported success of sacral and tibial neurostimulators offer tantalizing clues that successful treatment of urinary dysfunction may lie in the modulation of the brain–bladder axis, not the bladder muscle specifically (Burton et al., 2012; Siddiqui et al., 2010).

### 2.9 Altered cell compositions in the aging bladder

Changes in cell composition may contribute to dysfunction in the aging bladder. Single cell transcriptomics of the B6 mouse bladder found that aged animals exhibit nearly an 11% increase in the proportion of fibroblasts in comparison to both young or mature animals (Baker et al., 2021). Further, the same study found an increase in the ratio of mesenchymal to urothelial cells (Baker et al., 2021), a particularly important observation given that the epithelial‐to‐mesenchymal transition has been associated with tumor progression and a driver of poor prognosis in plasmacytoid urothelial carcinoma (Nomura et al., 2020). Another single cell bladder atlas reported that aging was associated with decreased mesenchymal and increased urothelial cell proportions (Tabula Muris, 2020), although these samples were denuded (detrusor muscle removed) which may explain this discrepancy.

### 2.10 Detrusor aging is associated with increased fibrosis, yet strength muscle strength is preserved

It is tempting to consider the old bladder as inherently weak; however, this view has been challenged based on evidence in murine models and human studies. In aging C57Bl/6 (B6) mice showing detrusor expulsive pressure is preserved and that muscle strength is not lost (Smith et al., 2012b). Deficits in detrusor contractility may be the result of maladaptive collagen recruitment, as fiber activation prematurely was associated with increased bladder wall stiffness in Fischer rats (Cheng et al., 2018). Multiple reports have identified thickening of the bladder wall and increased collagen deposition associated with aging in both humans (Lepor et al., 1992) and B6 mice (Hardy et al., 2019), though the relationship between fibrosis and bladder tissue dysfunction has not been demonstrated conclusively. Fibrosis in a rat model of bladder outlet obstruction is believe to be triggered through the NLRP3 inflammasome and increased IL‐1β production (Hughes Jr. et al., 2017), both of which are significantly upregulated in the aged bladder. The density of axons in the detrusor smooth muscle has also been reported to decrease with aging in humans (Gilpin et al., 1986). In B6 mice, expression of muscarinic and adrenergic receptors have been reported to decrease with aging (Kamei et al., 2018). Decreased receptor expression may provide insight into the decreased responses to excitatory and relaxatory stimuli seen in aged bladder strips from B6 mice (Hardy et al., 2019, 2021), Sprague–Dawley rats (Durlu‐Kandilci et al., 2015), and humans (Yoshida et al., 2001). Increased agrin—an essential element of the neuromuscular junction—is observed at the neuromuscular junctions of the developing rodent bladder (Gingras et al., 2005) and is required for neuromuscular junction integrity (Hoch, 1999). Improved understanding of how aging impacts the neuromuscular junctions in the bladder may further shed light on the structural changes that occur.

### 2.11 Mucosal aging and associated increases in inflammation, mitochondrial dysfunction, and cellular senescence

Aside from the detrusor, alterations in the mucosa (lamina propria + urothelium) are also observed with aging. Urothelial detachment, connective tissue thickening, and maladaptive morphologic changes have been observed in B6 mice (Schueth et al., 2016). Signaling changes of the urothelium, such as increased NLRP3 inflammasome expression, mitochondrial dysfunction, increased markers of cellular senescence (such as p21) and increased oxidative stress have also been reported in aged Sprague–Dawley rats (Chen et al., 2019). SIRT3, a protein involved in the elimination of reactive oxygen species, was also downregulated in aged Sprague–Dawley rats (Chen et al., 2019). In Fischer 344 rats, similar age‐dependent increases in p21 were observed, as well as cytochrome‐C (found in mitochondria, associated with increased cell death) and nitrotyrosine (associated with oxidative damage and apoptosis) (de Rijk et al., 2022). In urothelial primary cultures from aged Fischer 344 rats, impaired mitochondrial bioenergetics and decreased mitochondrial membrane potential were also reported (de Rijk et al., 2022). Alterations in purinergic signaling of the urothelium have been observed in aged B6 mice, with increased purinergic bioavailability being associated with increased voiding frequency and afferent firing (Daly et al., 2014). The interplay between the detrusor and mucosa is still being elucidated. Future investigations interrogating how aging impacts inter‐layer signaling are needed.

### 2.12 Aging is associated with increased heterogeneity of responses

**FIGURE 4:**
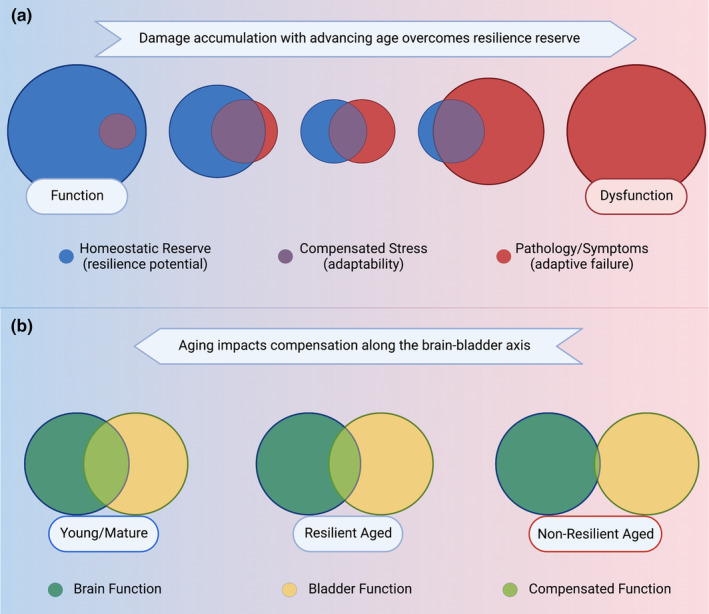
Homeostatic reserve diminishes with aging. (a) In robust/resilient organisms, stressors can be accommodated while still maintaining function (left). With advancing age, the likelihood of the organism to be able to compensate for these stressors diminishes while the instances of stressors end to increase demonstrated here by a shrinking blue circle (Homeostatic Reserve) and a growing red circle (Pathology/Symptoms). Organisms can compensate for stress while still maintaining function (purple), but aging increase the chance that stressors will fall outside of this adaptable range, resulting in dysfunction (right). (b) Compensation occurs along the brain–bladder axis, allowing for function to prevail despite the presence of stressors, particularly in young/mature (robust/resilient) organisms: The brain (teal) is able to accommodate the bladder (yellow) while still maintaining function (green), and vice versa (left). Aging diminishes how well one can compensate for the other, but in resilient organisms, function is still generally maintained despite the adaptive range (green) becoming smaller. In the case of non‐robust/frail organisms, certain conditions may preclude one end of the brain–bladder axis from compensating for the other (right). For example, a highly fibrotic bladder that is unable to contract cannot be compensated for by intact signaling from the brain—illustrated here by the barely overlapping green (right).

In many systems, it has been observed that aging increased the variability of responses to a particular stimulus when compared to young or mature animals, and the same has been observed in the lower urinary tract. Aging in and of itself is a heterogeneous process: the unique stressors effecting the individual paired with the underlying genetic risk for said individual results variable presentations. Maladaptive aging—that is, aging changes that preclude normal responses to stimuli—results in an increased risk of frailty or dysfunction that is unable to be accounted for in homeostatic mechanisms. Resilience is at the opposite end of this spectrum: Despite the increased accumulation of damage with age, response to stressors is relatively well tolerated and homeostatic mechanisms can compensate without exhibiting dysfunction (Figure 4) (Ferrucci et al., 2020).

Response heterogeneity has been previously associated with alterations in ion channel expression, as variances about mean responses to adrenergic stimuli in strip studies paralleled decreasing expression of *Hcn1*, encoding the hyperpolarization‐activated cyclic nucleotide‐gated channel 1 protein (Hardy et al., 2021), which has previously been shown to play an important role in adrenergic detrusor tension control with aging in B6 mice (Al‐Naggar et al., 2019; Lemtiri‐Chlieh et al., 2022; Mader et al., 2018; Singh et al., 2022). Apart from tissue‐level responses, heterogeneity is also exhibited in response to the stressor of cystometry. The majority of aging B6 mice respond to cystometric filling, developing pressure curves that increase in proportion to volume, and decrease when the bladder reservoir is emptied, only to cycle again as filling continues. However, a portion of aged mice—anywhere from 25% to 50%—fail to cycle during cystometric studies, instead slowly leaking volume with intravesicular pressure oscillating about a mean pressure in a sinusoidal‐like waveform (Hardy et al., 2019; Smith et al., 2012b). To date, it is not known what distinguishes the “responders” from “non‐responders,” though it is generally characterized as a multifactorial loss of resilience (Ramasamy et al., 2023). More molecular and mechanistic investigations will be necessary to identify if gene or protein expression changes are predictive of one phenotype over the other. Improvements to our understanding of the responder/non‐responder phenotype may be useful in human medicine, as there is little known what distinguishes aged individuals who develop urinary dysfunction from those who do not. Gene expression differences may prove to be the culprit behind these discrepancies.

## 3 CONSIDERATIONS FOR ANIMAL MODELS

### 3.1 Genetic diversity in aging studies

Shortcomings in our understanding of urinary aging have undoubtedly come from the limitations of our basic and translational research studies, the largest of which could be argued as the lack of genetic diversity in mouse urinary aging research. The most widely used mouse line in biomedical research is undoubtedly the B6 mouse. B6 animals are inbred, genetically identical mice that are widely available through commercial vendors. The utility of the B6 model is unfortunately also its downfall: The translation of many B6 studies to humans have had limited efficacy, as humans are genetically diverse and diseases/disease processes are often exacerbated by genetic predispositions. For example, a recent study investigating brain microstructure in mouse models of various genetic backgrounds found significant differences in connectivity and structure between the strains used (Wang et al., 2020). Lesions in the brain and spinal cord were found in the majority of 28 inbred mouse strains, though there were strain differences (Ward et al., 2022), emphasizing the need for the inclusion of genetic diversity in studies of aging. Continuing studies in B6 mice exclusively runs the risk that B6 animals are a biologic outlier and different from human biology. In the kidney, for example, certain strains exhibited increased susceptibility for dysfunction, while others (including B6) are more resistant (Bufi & Korstanje, 2022). The inclusion of other strains and stocks, particularly those of genetically diverse backgrounds, may better model the human condition.

### 3.2 Future therapeutic focus

In general, treatments for lower urinary tract dysfunction can be grouped into three categories: behavioral, surgical, and pharmacologic. Behavioral interventions can be exceptionally beneficial in limited circumstances: Limiting fluid intake in the evening hours, for instance, can help reduce instances of nocturia and timed voiding can reduce accidents in individuals with volume sensitivity or impaired cognition (Paraiso & Abate, 2009). Surgical interventions, such as sacral nerve stimulation devices, offer high rates of success in otherwise healthy adults, but risk–benefit must be considered in older adults as success is multifactorial (Katz et al., 2019). Decreased wound healing, tolerance for surgery/anesthesia, polypharmacy, and other comorbidities complicate how well an individual may recover and benefit from such a procedure.

Pharmacologic interventions—like anticholinergic and adrenergic drugs—are, as the name suggests, the next step in cases where behavioral interventions are insufficient to address the patient's symptoms (Abrams et al., 2006; Alhasso et al., 2005). However, these drugs have many side effects due to the nearly ubiquitous expression of cholinergic and adrenergic receptors across the organ systems of the body. Administration of these agents must be used cautiously, particularly in the geriatric patient, as dementia‐like side effects are not uncommon (Araklitis et al., 2020; Nishtala & Chyou, 2022). The lack of safe, efficacious treatments for older adults emphasizes the need to investigate novel therapeutic targets outside of the classic cholinergic/adrenergic sphere. Recent investigations have revealed several new targets outside of cholinergic and adrenergic mechanisms that could prove to be clinically beneficial.

Purine metabolism is gaining interest as a potential target for age‐related urinary tract dysfunction. Imbalances in purine metabolism are implicated in disease: Deficiency in the enzyme purine nucleoside phosphorylase (PNPase) leads to complications with immunity and neurodevelopmental processes (Grunebaum et al., 2020). Uroprotective purines inosine, adenosine, and guanosine are converted to ROS‐producing hypoxanthine and xanthine by PNPase (Birder & Jackson, 2022). Inhibition of PNPase with the partial inhibitor 8‐aminoguanine (8‐AG) “rebalances” the purine metabolome, preventing the conversion of uroprotective to urotoxic purine metabolites and reduces subsequent ROS production. In a study in Fischer 344 rats, administration of 8‐AG improved not only bladder function, but also induced structural changes: Aged bladders exhibited vascular tortuosity and reduced perfusion of the tissue; however, treatment with 8‐AG significantly improved blood flow and collagen fiber recruitment in aged rats to levels comparable to that of a young animal (Birder et al., 2020). Interestingly, PNPase inhibiton has also shown an effect on renal physiology. Administration with 8‐AG significantly increased sodium and glucose excretion while sparing potassium excretion in the urine, suppressing salt‐induced hypertension (Jackson et al., 2016).

The endocannabinoid system has been shown to regulate bladder sensory mechanisms, implicating modulation of this endogenous system as a potential therapeutic target (Andersson, 2016; Christie et al., 2021). Cannabinoid receptors CB1 and CB2 are expressed in the urothelium, as well as on neurons in the bladder wall (Tyagi et al., 2010). Activation of CB receptors in rodent models produces hyperalgesic affects against inflammation (Hedlund & Gratzke, 2016). Recent studies have supported a sensory mechanism of action for the role of endocannabinoid signaling in the bladder: Activation of CB receptors is believe to decrease firing of both low threshold A‐delta and high threshold C afferent fibers (Christie et al., 2021). These effects were observed in models of distension‐induced afferent firing (Walczak et al., 2009), as well as bladder pain syndrome models using ATP‐ or capsacin‐induced released of CGRP that were attenuated after administration of synthetic CB agonists (Hayn et al., 2008). These findings are supported by reports of improved symptoms with cannabinoid use in multiple sclerosis patients (Kim‐Fine et al., 2021). Patients reporting cannabis use within the past 3 months demonstrated double the odds for improvement in at least one bladder symptom (such as frequency, urgency, leakage/wetness, adult diaper usage, and bladder emptying), even when controlling for age and gender (Kim‐Fine et al., 2021).

## 4 CONCLUSIONS

The lower urinary tract is susceptible to the deleterious effects of aging, including both structural and functional alterations. Age‐associated lower urinary tract dysfunction stems from increased stressors impacting the adaptability of the brain–bladder axis. CNS changes, including brain atrophy, vascular deficits, and cellular dysfunction, contribute to impaired communication between the brain and bladder, which may directly contribute to dysfunction in both the cognitive and reflex control over micturition. In general, aged bladder tissue is less responsive to pharmacologic stimulation, exhibits significant changes in gene expression of key receptors, and is less able to accommodate stressors and maintain homeostatic norms. Aging is associated with increased variability of responses, demonstrating the need to better understand mechanistically what separates robust/resilient urinary tract aging from that of maladaptive aging. The incorporation of genetically diverse animal models into future studies may provide more relevant targets of interest to human medicine outside of the traditional adrenergic and cholinergic mechanisms. Urinary symptoms are highly prevalent in older adults with few efficacious options available therapeutically, demonstrating a clear and urgent need to address these knowledge gaps at the basic science level with animal models. Though new therapeutic targets have been identified, there is a need for clinical investigations to determine whether these targets will be beneficial to human medicine.

## AUTHOR CONTRIBUTIONS

Cara C. Hardy conceptualized and wrote this manuscript. Ron Korstanje aided in manuscript preparation and gave invaluable input for the direction and presentation of the topics covered in this text.

## CONFLICT OF INTEREST STATEMENT

The authors have no conflicts of interest to report.

## Data Availability

NA.
